# Age and sharing of needle injection equipment in a cohort of Massachusetts injection drug users: an observational study

**DOI:** 10.1186/1940-0640-8-20

**Published:** 2013-12-13

**Authors:** Katherine Tassiopoulos, Judith Bernstein, Edward Bernstein

**Affiliations:** 1 Department of Epidemiology, Harvard School of Public Health, 677 Huntington Ave., Boston MA, USA; 2 Department of Community Health Sciences, Boston University School of Public Health, 801 Massachusetts Ave., Boston MA, USA; 3 Department of Emergency Medicine, Boston University School of Medicine, Boston Medical Center, 1 Boston Medical Center Place, Boston MA, USA

**Keywords:** Injection drug use, Injection equipment sharing, Young age, Hepatitis C

## Abstract

**Background:**

Hepatitis C infection (HCV) among individuals aged 15–24 years has increased in Massachusetts, likely due to injection drug use. The prevalence of injection equipment sharing (sharing) and its association with age was examined in a cohort of out-of-treatment Massachusetts substance users.

**Methods:**

This analysis included baseline data from a behavioral intervention with substance users. Younger and older (<25 versus ≥25 years) injection drug users were compared on demographic characteristics, substance use practices, including factors present during the most recent sharing event (“event-level factors”), and HCV testing history.

**Results:**

Sharing was reported by 41% of the 484 individuals who reported injection drug use in the past 30 days. Prevalence of sharing varied by age (50% <25 years old versus 38% ≥25 years, p = 0.02). In a multivariable logistic regression model younger versus older individuals had twice the odds of sharing (95% CI = 1.26, 3.19). During their most recent sharing event, fewer younger individuals than older had their own drugs available (50% versus 75%, p < 0.001); other injection event-level factors did not vary by age. In the presence of PTSD, history of exchanging sex for money, or not being US born, prevalence of sharing by older users was higher and was similar to that of younger users, such that there was no association between age and sharing.

**Conclusions:**

In this cohort of injection drug users, younger age was associated with higher prevalence of sharing, but only in the absence of certain stressors. Harm reduction efforts might benefit from intervening on mental health and other stressors in addition to substance use. Study findings suggest a particular need to address the dangers of sharing with young individuals initiating injection drug use.

## Background

A recent *Morbidity and Mortality Weekly Report* (MMWR) found that, between 2002 and 2009, the rate of hepatitis C (HCV) infection increased statewide among young people (15–24 years of age) in Massachusetts, primarily among non-Hispanic whites. The investigators identified injection drug use as the primary risk factor for HCV infection in this population of adolescents and young adults, with heroin being the primary drug of injection [1]. During this same time period there was also an apparent increase in heroin use among young people in Massachusetts and other states [2].

In light of these recent findings, it is important to understand the connection between age and injection drug use and sharing of injection drug use equipment in particular. Shared use of injection drug equipment (sharing) is a strong risk factor for HCV infection [3]. While a few studies of injection drug users have found either no association with age and sharing [4,5] or have found that older age is a risk factor [6], most studies have reported that younger injection drug users are more likely than older users to share needles or other drug equipment [7-13]. The reasons for the observed association between younger age and sharing are multifactorial. New initiates into injection drug use do not have experience injecting and may thus require help from others [14]; they also may have not been adequately exposed to prevention messages about the risks of sharing [15]. Additionally, young users may learn to inject in the context of strong social networks that encourage sharing and where trust and friendship make sharing more comfortable and less risky [16]. What has not been previously explored is whether this association between younger age and sharing holds steady across all cohorts of injection drug users or whether there are factors that might modify this association, as is suggested by the seemingly contradictory findings mentioned above.

The overall objective of this study was to further the understanding of why younger individuals are often more likely to share injection drug equipment, and to identify factors that might modify this association. Toward this end we examined data from a cohort of Greater Boston area injection drug users followed during the period in which HCV infection in Massachusetts was observed to increase. Our primary aims were to: (1) describe demographic and drug use practices overall and by age (<25/≥25 years, to correspond to the upper age for which HCV infection was observed to increase in Massachusetts); (2) estimate the association between age and sharing, adjusting for other risk factors of sharing; (3) compare younger (<25 years) and older (≥25 years) individuals on factors present at their most recent sharing injection event; (4) explore whether the association between age and sharing varies between levels of factors indicative of personal stressors or social instability, including exchange of sex for money, post-traumatic stress disorder (PTSD) symptoms, and whether US born; and (5) summarize the self-reported HCV testing history and the HIV prevalence of injection drug users in the cohort, overall, by age group, and by sharing status.

## Methods

### Study sample

The parent study from which this analysis is drawn was a randomized controlled trial of a behavioral intervention to reduce sexual risk behaviors among hospital patients who reported drug use within the past 30 days. An ethnically diverse population of patients registered for medical care from November 2004 through May 2008 was screened for eligibility at an academic, urban, level-I, trauma center emergency department using a Health Needs History screening survey [17]. Eligibility criteria for the parent study included current (past 30-day) cocaine or heroin use, English or Spanish speaking, ages 18–54 years, and a Drug Abuse Severity Test Score ≥3 indicating moderate or higher severity [18]. In the current analysis, we included all enrollees from the parent study who reported injection drug use at the baseline visit. The study was approved by the Boston University Medical Campus Institutional Review Board, and written informed consent was obtained for all enrollees. A National Institutes of Health Certificate of Confidentiality was obtained to protect subject data from subpoena. Details of the enrollment process and intervention are described elsewhere [19].

### Study design and laboratory procedures

The present study is a cross-sectional analysis that used data collected at the baseline visit of the intervention study. Enrolled participants were interviewed face-to-face by trained research assistants for demographic characteristics, health status, drug use patterns, sexual practices, HCV testing history, and PTSD symptoms using the PTSD Checklist-Civilian Version (PCL-C) [20]. Testing for HIV infection was conducted using oral mucosal transudate (OMT) samples (OraQuick®, Orasure Technologies, Inc.) processed by the Massachusetts Department of Public Health Laboratory. The OMT specimens were tested for HIV antibodies by enzyme-linked immunosorbent assay (ELISA) and confirmed by western blot. Testing for HCV was not conducted.

### Exposure

The primary exposure of interest was age, dichotomized as younger (<25 years) or older (≥ 25 years). This definition of younger and older age was chosen *a priori* to correspond to the upper age for which hepatitis rates increased in Massachusetts (15–24 years of age) as described in the *MMWR*[1]. We also considered age as a categorical (18–25, >25-40, and >40 years) and a continuous variable in Aim 2 to further explore the association of age with sharing.

### Outcome

The outcome for Aims 2 and 4 was any disclosure of shared needles or other injection drug use equipment within the past 30 days based on responses to the question: “In the past 30 days when you injected drugs, how often did you use shared needles or works?” Responses varied from ‘never’, ‘sometimes’, ‘often’, and ‘always’. For these analyses, ‘any use’ was defined as a report of ‘sometimes’, ‘often’, or ‘always’ sharing.

### Covariates

We considered as potential confounders of the association between age and sharing (Aim 2) several risk factors for sharing identified in the literature, including: race; gender; education level (less than high school or high school and higher); current working status; living status (homeless, staying in shelter, or living temporarily with friends or family versus living in house, apartment, public housing, rooming house, halfway house, or group home); frequency of injection use in the past 30 days; specific drugs used in the past 30 days (injected or otherwise) (heroin, cocaine/crack, marijuana, PCP, speed/amphetamines, inhalants, barbiturates, benzodiazepines, hallucinogens); PTSD symptoms; history of receiving money in exchange for sex; and whether US born (defined as born in the US excluding Puerto Rico and other territories).

### Event-level factors

For Aim 3, we compared the prevalence of factors present during the most recent sharing episode (“event-level factors”) for younger and older injectors. In particular, we explored the type of drug shared, whether the individual had their own drugs to inject, the type of injection partner, whether the individual experienced withdrawal symptoms before injection, the setting of the sharing event, and where the individual obtained the needles used in the sharing event. These factors are important in defining the context of a recent sharing event; differences in the prevalence of these factors by age can help elucidate when and why younger and older individuals share.

### Effect modifiers

We also had an *a priori* interest in examining a subset of the covariates summarized above as potential effect modifiers of the association between age and sharing (Aim 4) because of their association with life stressors or social instability: history of receiving money in exchange for sex; PTSD symptoms; and whether US born.

### HCV and HIV testing

Participants were also asked whether they had ever been tested for HCV. If they answered ‘yes’, they were asked whether they had received their test results, and if so, whether the results were positive or negative. This information, and the results of HIV testing, were summarized for the group as a whole, both by age group and by sharing status (Aim 5).

### Statistical analysis

We summarized study sample characteristics, including demographics, drug using practices, HCV testing history, and HIV serostatus. We compared younger and older injection drug users by these characteristics using chi-square tests. Among those individuals who reported any sharing, we compared factors present during the most recent sharing event (event-level factors) for younger versus older users using chi-square tests, to explore differences in prevalence for younger versus older users who shared.

We used logistic regression models to estimate the association between age and sharing. A univariable model with dichotomous age as the sole predictor was first developed to provide an unadjusted odds ratio (OR) for the association of age with sharing. A multivariable model was then built to estimate the association of age and sharing, adjusting for confounding factors. Each potential confounder of the association between age and sharing was entered into the univariable model one at a time. When inclusion of a specific variable changed the OR for the unadjusted association between age and sharing by more than 10%, this variable was included in the final multivariable model as a confounder. Univariable and multivariable models with (a) continuous and (b) categorical age were also developed; the multivariable models included the covariates identified as confounders in the primary (dichotomous age) multivariable model.

To explore effect modification, we stratified multivariable models (which included covariates identified as confounders for Aim 2) of age and sharing by the categories of each potential effect modifier (history of receiving money in exchange for sex, PTSD symptoms, whether US born) to determine whether the association between age and sharing differed between these different categories. (For instance, we examined whether the association between age and sharing was different for those with PTSD symptoms and for those without symptoms). In those situations where there was qualitative evidence of effect modification (for example, no association observed in one category of the effect modifier but a strong association observed in another category), we created interaction terms between age and the covariate and included this term in the model to determine whether the interaction term was statistically significant.

## Results

One thousand thirty substance users were enrolled in the intervention study. Four hundred eighty-nine individuals (47.5%) reported having injected drugs within the past 30 days. Our analysis sample included the 484 injection-drug-using individuals (99%) who provided information on whether they had shared injection equipment within the past 30 days. One hundred one of the 484 individuals (20.9%) were <25 years of age (18.9-24.9 years), and 383 (79.1%) were ≥25 years of age (25.2-54.3 years).

Table 1 summarizes the demographic and behavioral characteristics of the study sample, stratified by age. Several factors differed by age. Fifty-one percent of younger users were female compared with 33% of older users (p < 0.01). Eighty-seven percent of younger users were white non-Hispanic compared with 53% of older users (p < 0.01). Frequency of cocaine use also differed by age, with twice as many older users reporting daily cocaine use (21.0% versus 10.1%, p = 0.01). The majority reported both daily use of heroin and daily injection drug use; these proportions did not differ by age.

**Table 1:** Characteristics of out-of-treatment injection drug users, by age (N = 484)

**Characteristic**	**Younger than 25 (N = 101) N (%)**	**25 or older (N = 383) N (%)**	**p-value**
**Female**	52 (51.5)	125 (32.6)	<0.01
**Race**			<0.01
Black	1 (1.0)	54 (14.1)	
White, non-Hispanic	88 (87.1)	204 (53.3)	
Hispanic	11 (10.9)	120 (31.3)	
Other	1 (1.0)	5 (1.3)	
**Born in US**	95 (94.1)	332 (86.7)	0.04
**Homeless**	45 (44.6)	195 (50.9)	0.26
**Education ≥ High School**	74 (73.3)	257 (67.1)	0.24
**Currently Working**	17 (16.8)	34 (8.9)	0.02
**PTSD Symptoms Score >50**	59 (58.4)	211 (55.2)	0.57
**Frequency of Cocaine/Crack Use**			0.01
No use	33 (33.3)	152 (39.9)	
1-3 times per month	22 (22.2)	57 (15.0)	
1-6 times per week	34 (34.4)	92 (24.1)	
Daily	10 (10.1)	80 (21.0)	
**Frequency of Heroin Use**			0.60
No use	5 (5.2)	10 (2.6)	
1-3 times per month	12 (12.5)	44 (11.6)	
1-6 times per week	11 (11.5)	50 (13.2)	
Daily	68 (70.8)	276 (72.6)	
**Frequency of Injection Drug Use**			0.86
1-3 times per month	15 (15.5)	56 (14.7)	
1-6 times per week	14 (14.4)	48 (12.6)	
Daily	68 (70.1)	277 (72.7)	
**History of Exchanging Sex for Money**	26 (25.7)	93 (26.5)	0.92
**Any Needle Sharing, past 30 days**	**52 (51.5)**	**146 (38.1)**	**0.02**

The following variables had missing observations: PTSD symptoms (n = 1); cocaine/crack frequency (n = 4); heroin frequency (n = 8); injection frequency (n = 6).

One hundred ninety-eight of 484 (40.9%) injection drug users reported having shared within the past 30 days. This proportion differed by age: approximately 50% of individuals <25 years reported sharing compared with 38% of those ≥25 years (p = 0.02).

The unadjusted OR for the association between younger age and sharing was 1.72 (95% confidence interval [95% CI] = 1.11, 2.68). In the final multivariable model, younger injection drug users had twice the odds of sharing of older users (95% CI = 1.26, 3.19) (Table 2). Variables that were included in the final model as confounders of the association between age and sharing included frequency of cocaine/crack use, injection frequency, and whether US born. Those who reported using cocaine or crack daily had 2.75 times the odds of sharing of those who did not use cocaine or crack. Those who were born outside the US had twice the odds of sharing of those born in the US. Injection frequency was not associated with sharing in the multivariable model. Gender, education level, and homelessness did not confound the association between age and sharing. The adjusted OR for continuous age and sharing was 0.96 (95% CI = 0.94, 0.98). The adjusted OR for individuals >25-40 years (versus 18–25 years) was 0.61 (95% CI = 0.38, 0.99), and for individuals older than 40 years (versus 18–25 years) was 0.29 (95% CI = 0.16, 0.53).

**Table 2:** Association between age and sharing of injection drug equipment (N = 484)

**Characteristic**	**Unadjusted model**	**Multivariable model**	**P-value**
**Age in years**			**0.004**
<25	1.72 (1.11, 2.68)	1.99 (1.25, 3.18)	
≥25	ref	ref	
**Frequency of Cocaine/Crack Use**			0.002
Daily		2.75 (1.62, 4.68)	
1-6 times per week		1.87 (0.90, 3.03)	
1-3 times per month		1.61 (0.90, 2.85)	
No use		ref	
**Born outside US**		2.09 (1.16, 3.79)	0.02
**Frequency of Injection Drug Use**			0.24
Daily		1.59 (0.90, 2.82)	
1-6 times per week		1.73 (0.82, 3.62)	
1-3 times per month		ref	

Frequency of cocaine/crack use, born outside US, and injection frequency are included in the multivariable model as confounders of the association between age and injection equipment sharing.

Sharing is defined as any sharing of injection drug equipment in the past 30 days.

Table 3 summarizes the event-level factors present during the most recent sharing event for the 198 individuals who reported sharing, stratified by age group. A significantly lower proportion of younger than older individuals said that they had their own drugs available for injection (50% versus 75%, p < 0.001). Most individuals (77%), regardless of age, reported using heroin during their most recent sharing event, and the majority also reported experiencing withdrawal symptoms prior to injecting. A somewhat lower proportion of younger than older users reported injecting either at their home or that of a friend, but this difference was not statistically significant. A higher proportion of younger than older users reported being with a sexual partner (42% versus 27%), but overall the difference was not statistically significant. Most individuals obtained their needles at either a needle exchange or pharmacy.

**Table 3:** Event-level characteristics at most recent drug injection equipment sharing event (N = 198)

**Event-level characteristic**	**Younger than 25 (n = 52) n (%)**	**25 or older (n = 146) n (%)**	**P-value**
**Had Own Drugs**	26 (50.0)	109 (74.7)	0.001
**Type of Drug Injected**			0.50
Heroin	42 (80.8)	110 (75.3)	
Cocaine	4 (7.7)	7 (4.8)	
Heroin and Cocaine	6 (11.5)	28 (19.2)	
Other	0 (0)	1 (0.7)	
**Injection Setting**			0.20
Shooting Gallery	2 (3.9)	7 (4.8)	
Home/Friend’s Home	21 (41.2)	80 (54.8)	
Public Place	28 (54.9)	59 (40.4)	
**Injection Partner**			0.22
Sexual Partner	22 (42.3)	40 (27.4)	
Friends	19 (36.5)	74 (50.7)	
Stranger(s)	2 (3.9)	7 (4.8)	
Alone	9 (17.3)	25 (17.1)	
**Where Obtained Needles**			0.36
Needle Exchange	25 (49.0)	87 (60.0)	
Injection Partner(s)	7 (13.7)	22 (16.2)	
Pharmacy	18 (35.3)	35 (34.1)	
Found	1 (2.0)	1 (0.7)	
**Withdrawal Symptoms before Injection**	40 (76.9)	111 (76.0)	0.90

Missing observations: Setting=1; Where Obtained Needles=2.

The association between age and sharing differed between categories of each potential effect modifier, including PTSD positivity, receipt of money for sex, and whether US born (Table 4). Specifically, among individuals with a PTSD symptom score ≤50 (indicating fewer PTSD symptoms), younger individuals had four times the odds of sharing of older individuals, while among those with a PTSD symptom score >50, there was no association between age and sharing. Similarly, among those with no history of receiving money for sex, younger individuals had more than twice the odds of sharing of older individuals, but no association was observed among those with a history of receiving money for sex. For those born in the US, there was a strong association between younger age and sharing, but among those born outside the US, there was no association between age and sharing.

**Table 4:** Effect modification of the association between age and sharing of injection drug equipment

**Age, yrs (n)**	**% Share**	**OR (95% CI)**	**Age, yrs (n)**	**% Share**	**OR (95% CI)**	**p-value for interaction term**
**PTSD Symptoms Score ≤50 (n = 213)**			**PTSD Symptoms Score >50 (n = 270)**			
**<25: n = 42**	59.5%	4.07 (1.89, 8.77)	**<25: n = 59**	45.5%	1.29 (0.69, 2.39)	0.09
**≥25: n = 171**	30.4%	ref	**≥25: n = 211**	44.6%	ref	
**No History of Money for Sex (n = 332)**			**History of Money for Sex (n = 119)**			
**<25: n = 74**	55.4%	2.67 (1.52, 4.66)	**<25: n = 26**	42.3%	0.89 (0.34, 2.34)	0.05
**≥25: n = 258**	34.5%	ref	**≥25: n = 93**	48.4%	ref	
**Born in US (n = 427)**			**Born outside US (n = 57)**			
**<25: n = 95**	53.7%	2.37 (1.46, 3.86)	**<25: n = 51**	16.7%	0.15 (0.01, 1.99)	<0.01
**≥25: n = 332**	34.6%	ref	**≥25: n = 6**	60.8%	ref	

N=33 missing information on receipt of money for sex.

Models are adjusted for frequency of cocaine injection, frequency of injection drug use, and (except for model of effect modification by US born) whether US born.

Sharing is defined as any sharing of injection drug equipment in the past 30 days.

Among those without PTSD, 60% of younger and 30% of older users shared (p < 0.01), while among those with PTSD, 45% of both age groups reported sharing (p = 0.87). Among those not reporting exchange of money for sex, a significantly higher proportion of the younger injection drug users reported sharing (55% versus 35% [p < 0.01]); but among those who said they had received money in exchange for sex, about 50% of both younger and older individuals reported sharing (p = 0.58). We observed a similar pattern for place of birth: among those born in the US, younger individuals were more likely than older to report sharing (54% versus 35%, p < 0.01), while among those not born in the US, there was greater reporting of sharing among older users (61% versus 17%, p = 0.04), although the numbers in these cells were very small.

Overall, 77% of the study sample reported having been tested for HCV, and there was no difference in testing by age (p = 0.22). Most individuals (95%) received their test results, again with no difference by age (p = 0.30). Among those who received their HCV test results, younger individuals were somewhat less likely to report having tested positive (62% versus 71%), but this difference was not statistically significant (p = 0.33). There was also no difference in HCV testing patterns between those who reported current sharing and those who did not (p = 0.66); however, among those who received their test results, those who reported sharing injection equipment were significantly more likely to report testing positive (80% versus 62%, p < 0.01).

Ten percent of those 25 years and older, and 5% of those younger than 25 years, tested positive for HIV (p = 0.17). The prevalence of HIV infection did not differ between those reporting shared use of injection equipment and those reporting no sharing (7.6% versus 10.5%, p = 0.43).

## Discussion

In this paper, we describe a cohort of injection drug users whose primary drug of choice was heroin. The demographic makeup of the cohort varied by age, with individuals <25 years old more likely to be female and white non-Hispanic. The cohort was comprised of individuals with fairly severe substance use, with the majority reporting daily injection use and daily heroin use. In addition, most reported experiencing withdrawal symptoms prior to their most recent injection equipment sharing event.

Injection drug use frequency did not vary by age; however, sharing of injection equipment did vary by age, with individuals <25 years old significantly more likely to share than individuals ≥25 years old. Our findings are consistent with those of most previous studies that examined the association between age and sharing. This association remained strong after adjusting for several risk factors of sharing including frequency of cocaine use, frequency of injection, and whether US born. We did not have information on age at initiation of injection drug use. However, it is reasonable to propose that younger individuals in our cohort on average initiated injection drug use more recently than did older individuals. Since people are often introduced to injection drug use by other users, one reason for the association between younger age and sharing may be that younger, newer users still rely on others’ help when injecting [14]. Our finding that younger individuals who share were more likely than older individuals to report that they did not have their own drugs to inject is consistent with this reason. Use and abuse of prescription opioids is also high among young individuals and has emerged as an important introduction to heroin use and heroin injection [21]. It is believed to be one of the reasons for the recent increase in injection drug use and HCV infection in young adults observed in Massachusetts and elsewhere [22].

While younger age was, overall, a strong risk factor for sharing, we found this association differed qualitatively depending on other factors that may be related to personal stressors or social instability. To our knowledge, this is the first study to have examined and identified potential effect modifiers of the association between age and sharing. Specifically, we found that the effect of younger age on sharing was substantial and significant among individuals who were born in the US, who did not have a history of receiving money for sex, or who did not have symptoms of PTSD. However, among those with one or more of these stressors, younger age was not associated with sharing. While we emphasize that these effect modification analyses and results are exploratory, they do suggest that, in the presence of other destabilizing factors in the lives of injection drug users, sharing is more prevalent among all ages, and the relative importance of younger age as a risk factor for sharing is diminished.

We found evidence that, in the presence of these stressors, the prevalence and frequency of sharing by older users was greater and more similar to that of younger users, than in the absence of these stressors. Previous research has found that older users or individuals with more extensive injection drug use experience are less likely to share than are younger users, either because they have learned how to inject on their own or because they have learned the health risks of sharing injection equipment [23]. However, it could be that in the chronic presence of stressors such as PTSD or exchange sex, individuals are not able to develop the capacity to reduce their frequency of sharing as they get older. It may also be that individuals with these stressors have less stable or more frequently changing social networks, something which has also been associated with more risky drug using behavior [24,25]. Factors specific to the social networks or life stressors of injection drug users may in part explain the conflicting results of previous studies regarding age and injection equipment sharing. For example in Stein et al. [5], where age was not significantly associated with sharing, all study participants met *DSM-IV* criteria for major depressive disorder, dysthymia, or persistent substance-induced mood disorder.

Our results revealed greater reporting of sharing by those born outside of the US. Most of the individuals in our cohort who were born outside of mainland US were born in Puerto Rico. Zerden et al. [26] recently showed that Puerto Ricans born and residing in Puerto Rico were more likely to share needles than were Puerto Ricans born in the US and residing in Massachusetts.

Previous research has found that young injection drug users have lower rates of HCV testing than older users [27]. We did not observe the same finding in our cohort. This may be due to the fact that these individuals were identified in a clinical setting with minimal financial barriers to care, and may, therefore, have had more access to health care and testing services than other populations of injecting drug users.

It is worth commenting that syringe and needle exchange and legal access to over-the-counter purchases have been successful public health strategies in Massachusetts; however, in our study, the majority of individuals who reported sharing said the equipment used in their most recent sharing event was obtained at either needle exchange or pharmacy, and most individuals who were tested for HCV reported testing positive. Access to clean equipment for these individuals has, therefore, not translated into sufficient protection against HCV infection. Strategies to address the precursors of sharing are critically needed.

Our study has several limitations related to the cross-sectional nature of the analyses. The lower prevalence of sharing observed for older users might not be due to safer injecting practices, but may instead be due to an overall reduction in substance use or in injection frequency over time, thereby reducing the opportunity for sharing. However, in our cohort, the frequency of injection drug use as well as the frequency of heroin use (the drug most individuals reported injecting) did not differ for older and younger users. Alternatively, the lower prevalence of sharing could be due to a survivor effect, where older individuals with years of sharing injection drug equipment do not survive and are not represented in our cohort. We are not able to assess this likelihood in the present study.

We did not have information on age at initiation of injection drug use and, therefore, cannot confirm that younger users initiated injection drug use more recently than older users. We did not collect information on the social networks of our participants; the potential influence of social networks on the association between age and sharing should be specifically assessed in future investigations. Our examination of effect modification was exploratory, as the study was not designed to examine effect modification and was, therefore, not optimally powered to identify statistically significant modifiers. Future studies with larger study samples should examine the same and additional effect modifiers related to social stress and instability, such as stressful life events and social support.

Also, since our cohort was comprised of injection drug users registered for health care at a busy urban emergency department, they are not necessarily representative of all injection drug users in Massachusetts. We did not conduct HCV testing and instead relied on self-reported information on HCV testing practices, which may mean that the observed prevalence of HCV testing was subject to recall bias. However, we did conduct HIV testing, and HIV test results were highly consistent with individuals’ self-reports of their HIV serostatus, suggesting that biased reporting of HCV testing patterns was minimal (data not shown).

## Conclusions

Most of the individuals in our cohort, regardless of age, reported daily heroin use and daily drug injection. Many of the individuals, again regardless of age, reported symptoms of PTSD, were homeless, or were not currently working, and older individuals reporting these stressors were more likely to share. Therefore, our findings suggest that harm-reduction efforts for these individuals should incorporate not just efforts to reduce drug injection and sharing of drug equipment, but also should address larger disruptive aspects of these individuals’ lives—such as mental health and social instability. In addition, as other investigations have pointed out, prevention and harm-reduction efforts for younger individuals who have just begun to inject should continue to ensure that these individuals are knowledgeable regarding the risks of sharing [28,29].

## Competing interests

The authors declare that they have no competing interests.

## Authors’ contributions

KT: Corresponding author, who performed the statistical analysis, interpreted the data and led the writing and editing. EB: Principal investigator, who designed and conducted the study, trained coders, reviewed and offered substantive critiques and revisions to manuscript drafts throughout the process. JB: Co-principal investigator, who designed and conducted the study, trained coders, reviewed and offered substantive critiques and revisions to manuscript drafts throughout the process. All authors read and approved the final manuscript.
